# Patient and Caregiver Perspectives on an eHealth Tool: A Qualitative Investigation of Preferred Formats, Features and Characteristics of a Presurgical eHealth Education Module

**DOI:** 10.1177/11795727211010501

**Published:** 2021-04-21

**Authors:** Holly Reid, Somayyeh Mohammadi, Wendy Watson, Julie M Robillard, Morag Crocker, Marie D Westby, William C Miller

**Affiliations:** 1 Department of Occupational Science and Occupational Therapy, Faculty of Medicine, University of British Columbia, Vancouver, British Columbia, Canada; 2 Rehabilitation Research Program, GF Strong Rehabilitation Centre, Vancouver, British Columbia, Canada; 3 Vancouver Coastal Health, Vancouver, British Columbia, Canada; 4 Centre for Hip Health and Mobility, Vancouver, British Columbia, Canada; 5 BC Children’s and Women’s Hospital, Vancouver, British Columbia, Canada; 6 Division of Neurology, Department of Medicine, University of British Columbia, Vancouver, British Columbia, Canada

**Keywords:** Hip replacement, knee replacement, eHealth, osteoarthritis, online tool, qualitative study, prehabilitation

## Abstract

**Introduction:**

Total hip and total knee replacement (THR and TKR) are suggested for reducing joint pain resulting from hip and knee osteoarthritis (OA), especially when other interventions have not resulted in desired outcomes. Providing prehabilitation education can improve patients’ psychological and physical well-being before and after surgery. The use of electronic health (eHealth) tools can be considered an effective method to increase patients’ access to prehabilitation, particularly for those facing barriers to attending diagnosis-specific in-person education sessions. However, limited attention is paid to both caregiver and patient perspectives regarding the delivery formats, features, and characteristics of eHealth tools.

**Method:**

Patients with hip (n = 46) and knee OA (n = 14) and their family caregivers (n = 16) participated in in-person focus groups or phone interviews. Participants were shown a mock-up of an eHealth module, and asked to share their preferences regarding the formats, features, and characteristics of the eHealth prehabilitation tool. Data was transcribed verbatim and coded using primary thematic and secondary content analyses.

**Result:**

Analyses revealed 3 main themes: 1. “easier to understand” emphasizes patients’ preferences on delivery formats and features; 2. “what does that mean?” highlights requests for clear and simple information; and 3. “Preparation, right?” shows patients’ perspectives on the best time to have access to the eHealth tool.

**Discussion:**

Participants’ preferences for prehabilitation tools included offering eHealth tools in multiple mediums of delivery (eg, written materials, pictures, videos). Participants preferred simplified information that emphasized the key points and rationale for the knowledge. There were differences in preferred timeline for having access to prehabilitation education, such as some participants wanting to receive prehabilitation well in advance, while others stated just before surgery was adequate. Our findings provide novel and actionable information about patient and caregiver perspectives on features and characteristics of prehabilitation education for patients with hip and knee OA.

## Introduction

Total hip replacement (THR) and total knee replacement (TKR) are among the most effective ways to reduce joint pain and improve function in individuals with advanced hip and knee problems, most commonly resulting from osteoarthritis (OA).^1^ In 2018 to 2019 more than 62 000 hip replacement and 75 000 knee replacement surgeries were conducted in Canada. These replacement surgeries cost the healthcare system more than $1.4 billion per year for inpatient hospital and physician costs.^2^ The total cost of total joint replacement (TJR) surgeries is underestimated, as there are rehabilitation, travel, education, and additional out-of-pocket costs to the patient and medical system.^2^

Health promotion interventions such as prehabilitation (education and exercises received by patients before surgery) are becoming increasingly vital as they reduce direct and indirect health care costs,^3^ and improve patient care and recovery.^4-6^ Prehabilitation includes education related to the surgery the patient will undergo including information on preparing for surgery, the surgical procedure, and the expected outcomes.^5-7^ However, the depth and consistency of this information varies greatly depending on the source of the prehabilitation content that the patient has access to. Prehabilitation has the potential to improve outcomes for both the patient and the healthcare system. For instance, evidence suggests there are reduced costs of hospitalizations for patients who receive prehabilitation and by informing patients what to expect before, during and after surgery, prehabilitation education can result in lower levels of preoperative anxiety.^4-6^ In addition, patients who received prehabilitation have better pain management skills.^7^ Therefore, to enhance recovery and reduce indirect costs, it is essential to provide prehabilitation for patients undergoing hip and knee replacement surgery and their family caregivers.^8^

Prehabilitation is commonly delivered through traditional methods such as in-person sessions or written materials. However, over the past few years, the use of electronic health (eHealth) which can be defined as the use of information and communication technologies in delivering educational materials to patients has increased substantially.^9,10^ eHealth programs “have the potential to support care delivery models, engage patients, and deliver self-assessment and self-management tools.”^11^ Specifically, eHealth has been shown to improve quality of care for older adult patients,^12^ enhance communication between patients and health care providers, reduce costs, and increase access to health care for underserviced rural and remote communities using evidence-based health information.^13^ Therefore, an opportunity exists to use eHealth technologies with patients who are preparing for THR and TKR.

In order for eHealth to be effective, particularly with older adults, it is necessary to integrate patients’ preferences regarding educational formats, features, and overall characteristics of an eHealth program.^12,13^ Investigations regarding TJR patient preferences toward eHealth, show that patients prefer easy tap interfaces, progress reports and knowledge tips.^14^ However, so far, when developing eHealth tools for patients, especially patients with THR and TKR, the perspective of patients and their family caregivers was not included in these studies and remains a gap.

To make eHealth interactive and successful, a variety of design concepts, parameters, and features should be used.^15^ Studies on education needs for patients undergoing THR and TKR showed patients preferred multi-modal education delivery and had an interest in accessing health applications or technologies.^16^ In addition, other factors, such as increasing the learners’ control over the educational materials, and tailoring the content based on the learners’ needs, increase the interactivity of eHealth programs.^17^ However, in developing eHealth tools, often only researchers and instructional designers are involved, and end-users’ (ie, patients) opinions are overlooked or are only assessed after eHealth tool development is completed (eg, in refs.^18-20^). It is essential to investigate both patients’ and family caregivers’ perspectives and preferences regarding eHealth tools prior to developing them. Including caregivers’ input regarding prehabilitation education is important, as they play a key role in the uptake and implementation of pre-surgical education the patient receives. Therefore, the *purpose* of our study is to explore and understand patients’ and family caregivers’ perspectives and preferences in delivery format, features, and characteristics of an eHealth tool aiming to provide prehabilitation for patients undergoing THR and TKR.

## Methods

This is a single qualitative study using focus groups and individual interviews with 2 samples. Sample 1 included patients with hip OA and their family caregivers while sample 2 included patients with knee OA. Caregivers were not included in the sample of participants with knee OA. We recruited participants through advertisements and pamphlets posted in several local hospitals in 1 health region of the province of British Columbia (BC) in Canada. Participants were also recruited through email invitations sent by the OsteoArthritis Service Integration System (OASIS), which provides in-person education for patients undergoing joint replacement in some parts of BC and the Vancouver Health Centre Research Institute. Interested individuals were invited to contact our research center. After receiving a call from an interested individual, a research assistant in our center provided more information about the study and checked their eligibility. Eligible individuals were sent a consent form via mail or email, based on their preference, and were asked to sign/e-sign the consent form and send it back to the research assistant. All participants had at least 24 hours to read and review the consent form before taking part in the interview.

### Eligibility criteria

Eligibility criteria for participants with hip or knee OA were being 45 years or older and having recently had or being on a waitlist for THR or TKR surgery and were comfortable with speaking and reading in English. Participants with hip OA were asked to invite one of their family caregivers. There were no inclusion/exclusion criteria for family caregivers. Exclusion criteria included patients having rheumatoid arthritis or other forms of inflammatory arthritis.

### Data collection

#### Focus groups and semi-structured interviews

Participants with hip OA and their family caregivers were assigned to 1 of the 9 focus groups (2-9 participants per group) which took place between November 2017 and March 2018 in BC. There were 5 phone interviews with individuals who could not participate in the focus groups due to time restrictions or mobility limitations. Participants completing the phone interview were sent a link to the mock-up module prior to the interview, and the researchers and participant went through the mock-up in real-time to discuss the same questions asked of the focus group and in-person interviews. The focus groups and interviews consisted of 2 parts. Part 1 focused on the preferred content and learning preferences of patients with hip and knee OA regarding the prehabilitation education (ie, Mohammadi et al., submitted). Part 2 (presented in this paper) centered on the participants’ preferences in delivery format (eg, texts, videos, voiceovers) and characteristics of a mock-up of an eHealth tool delivering prehabilitation education. Focus groups and interviews lasted up to 90 minutes, including a short break after part 1. The interview guide (Table 1) outlines the questions and prompts used for the interviews and focus groups.

**Table 1.** Semi-structured interview for assessing patients’ preferences regarding formats, features, and characteristics of eHealth programs.

Introduction	
There are many different ways of delivering health education, such as group sessions, one on one, reading etc. Now we will be looking at online education. The actual online education modules we will create will include seven sections. Right now, we are just going to work through one of the sections of an online education module, to get your feedback.	
1. Learning objectives	The section we’ll look at today is about Hip [knee] Precautions, or movement that you cannot do following your hip [knee] replacement surgery. We will give you the information using four different formats and see what you like best.
2. Hip precautions- text only [or knee precautions]	The first format is Text Only.
	So, I will give you a minute to read over this.
	– Are there any terms that aren’t clear. For example, is it clear what is meant by not bending your hip past 90 degrees?
3. Hip precautions- images with text [or knee precautions]	Now, we’ll look at the same information about hip precautions using text and images.
	– What are your thoughts on this format?
	Is it clear? What do you think this image is trying to tell you?
	– For example, do the images help with understanding of what it means to not bend your hip past 90°? Or was text alone enough to understand that
4. Hip precautions- images with voice over [or knee precautions]	Now, we’ll look at the same information about hip precautions using text, images, and a voice over.
	– What are your thoughts? Does the voice over add any value to the education? Would you use the voice over?
5. Hip precautions- video [or knee precautions]	Now, we’ll watch a video describing the hip precautions.
	– What are your thoughts? Is this more clear than the text and images examples? Would you like the option to have both? For all sections or just some sections?
6. Hip Precautions- white boarding video [or knee precautions]	Now we’ll watch another video, something known as “whiteboard animation”. We’ll just look at a short bit of this clip
	– What are your thoughts? Would you find this type of format helpful? How does it compare to the previous video?
Feedback questions	
Okay, to review, we’ve looked at information about hip [knee] precautions in four different formats (Text only, text and images, text and images and voice over, and video).	
How do the different formats compare?	
*Prompt*: Were any of these formats alone enough to learn about hip [knee] precautions?	
*Prompt*: Which is your preference? Why?	
Would you like to see a combination of formats?	
How do these formats compare to receiving written material, which is current practice?	
Do you still feel that you would want an in-person education session after viewing this material?	
QUIZ	
Thank you for your feedback about that particular section. We now want to get your feedback about potential quiz questions, which could be used throughout the education module to assess your learning. We want your feedback on whether you find quiz questions useful, and which type of questions you would prefer.	
1. Quiz 1 (yes/no)	The first format for quiz questions is a Yes/No format.
	– Is the question clear? Would it be helpful to have questions like this throughout the education module?
2. Quiz 2 (multiple choice)	Now I’ll show you another format for a quiz question, multiple choice.
	– What do you think of this format?
3. Quiz 3 (drag and drop)	This is a drag and drop format, what do you think?
Now we will take a look at one more quiz question, which is related to content we have not covered today – Seat height section. If you can, focus on the format of the question rather than the material that it covers.	
4. Quiz 5 (seat height) order questions	Now I’ll show you another format for a quiz question, checkboxes. What do you think of this format?
QUIZ feedback questions	
To review, we looked at 4 different formats for quiz questions – yes/no question, multiple choice, drag and drop, and order questions. Do you have a preference for the type of quiz question? Was the format of the quiz questions clear?	
Do you have a preference for images to be added in these questions? Would these sorts of questions add to your learning?	
Resource page	This is the last page, which includes links to resources. Would you click on the links?
	The resources and checklist can be downloaded and then printed. For example, this is the checklist (Click on it). Is this something that you would print off? Would you like a checklist or action plan that you can complete online and then print?
	Would you print the Before, During and After Hip Replacement booklet from this page?
	Is there anything else you would like on a conclusion page?
Conclusion	Thank you for all of your feedback about the online education. We just have a couple of concluding questions for you.
	How far ahead of your surgery would you want online information made available to you? For example, right after being placed on waitlist, 3 mo before your surgery, etc.)
	Who else should this resource be made available to? that is, family doctor
Member checking	I’ll summarize what I heard today, 3-4 key points, how does that sound?
Thank you	Thank you again for your feedback; it will be helpful in creating educational content.

Participants in the knee OA group took part in an in person or over the phone semi-structured interview (based on their availability and geographical location). Interviews followed the same format as described above. Only semi-structured interviews were conducted with patients with knee OA to facilitate the data collection procedure and reduce the expenses associated with the data collection.

**Figure 1.**
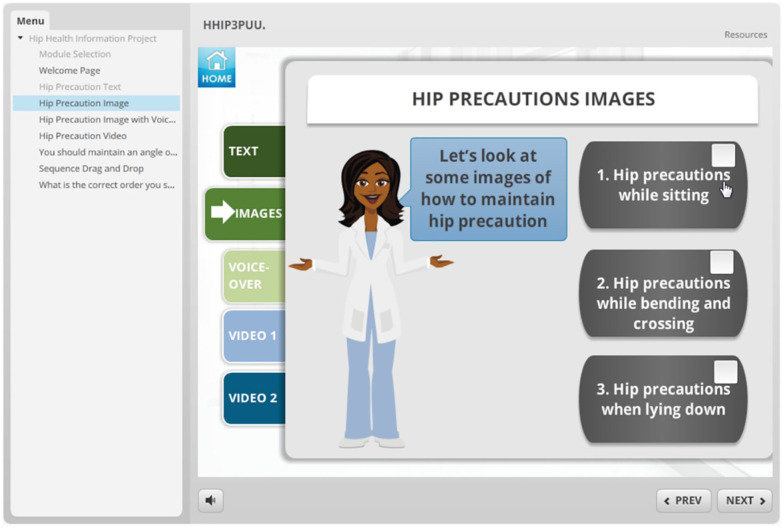
Screenshot of the eHealth mock-up that was used in the hip OA group.

**Figure 2.**
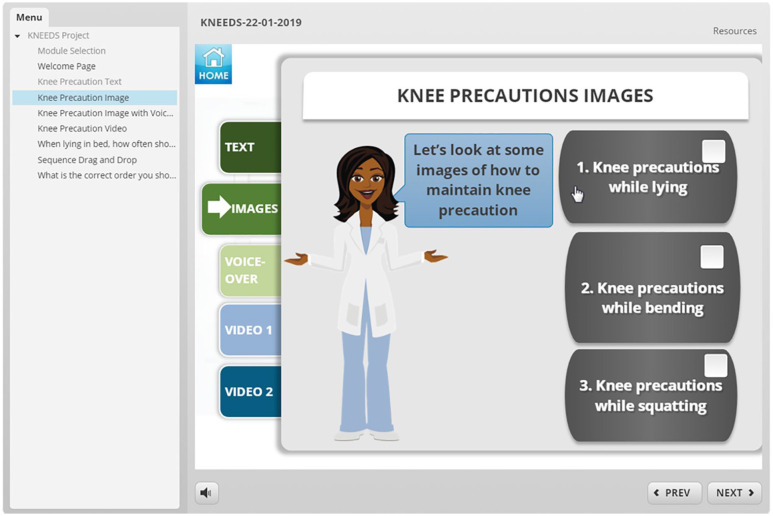
Screenshot of the eHealth mock-up that was used in the knee OA group.

**Figure 3.**
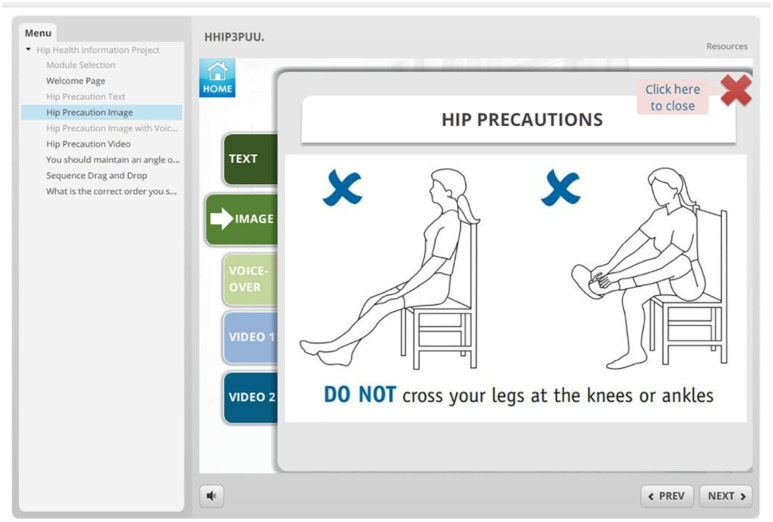
Screenshot of the eHealth mock-up that was used in the hip OA group.

**Figure 4.**
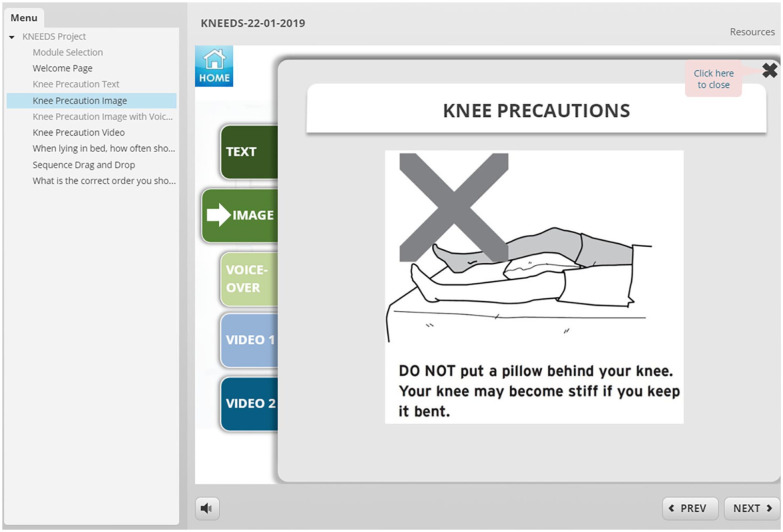
Screenshot of the eHealth mock-up that was used in the knee OA group.

**Figure 5.**
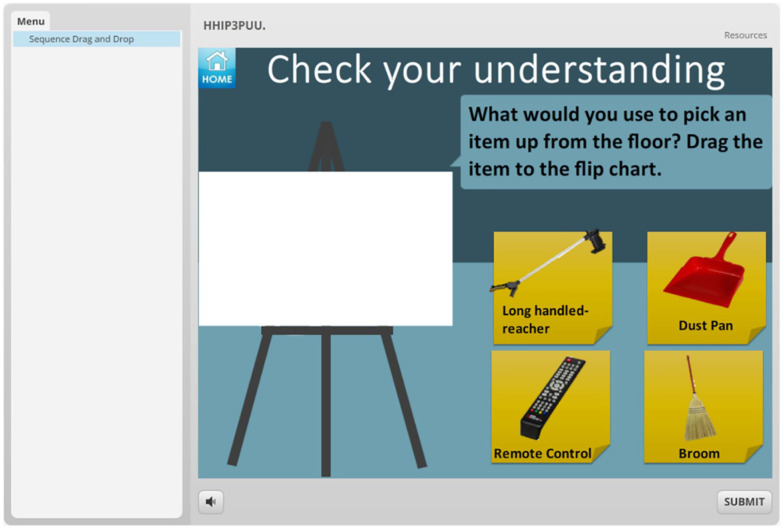
Snap shot of the eHealth mock-up that was used in the hip and knee OA group.

**eHealth module:** To investigate patients’ preferences in delivery format, features, and characteristics of an eHealth tool, we developed 2 mock-ups of an online module using Storyline 2.^21^ One mock-up module was used in our interviews with patients with hip OA and the other was used in the interviews with patients with knee OA. The formats were similar; however, the content, images, and videos reflected the specific diagnoses. These mock-ups served as an example and consisted of different sections. (Figures 1–5 provide examples). Participants were informed the focus was on their comments on the formats and not on the content of the examples (See Table 1, Part 2 questions and prompts). The mock-ups aimed to elicit participants’ perspectives on presenting information using written materials, images, voiceovers, different video styles (ie, short videos of actors/actresses presenting information and whiteboard videos), different formats of quizzes (ie, yes and no or true and false, multiple choice, drag and drop, ordering), and resource pages. All participants provided informed written consent, and the study was approved by The University of British Columbia ethics committee (knee OA sample (KNEEDS): H18-01417; Hip OA sample (HHIP): H15-03410).

### Research team and reflexivity

#### Researcher characteristics

HR is an occupational therapist with clinical experience in private practice, acute care and community settings. They have training and experience in collecting and analyzing qualitative data. SM has a Ph.D. in health psychology, with training as a clinical counsellor and health psychologist and had 3 years experience conducting research on patients with hip and knee OA at the time of this research. All authors have at least a Bachelor level education or higher (SM, JMR, MW, WCM), and experience as a clinician or researcher within the context of arthritis and TJR care. Six of the authors are female, and 1 is male (WCM). All reside and work within Western Canada.

Researcher reflexivity included field notes to record biases and emerging understanding. Researchers with different backgrounds including occupational therapy, physical therapy, health psychology and medicine were involved in the data collections and analyses which provided an opportunity for researcher triangulation. At the end of each semi-structured interview or focus group, the interviewer provided a summary of the interview and invited participants to verify whether the provided summary is accurate.

### Data analyses

All data was transcribed verbatim and then imported into excel files. Participants selected or were assigned a pseudonym for each interview or focus group and all identifiers were recorded and kept separate from transcripts. Primary thematic analyses and secondary content analyses were used to investigate patients’ preferences regarding the eHealth mock-up.^22^ When used in collaboration with primary thematic analysis to develop themes, secondary content analysis plays a supportive role to strengthen data analysis.^23^ Data are strengthened as a result of the interplay of these approaches, as content analysis identifies code frequency which can be used in theme development, while thematic analysis honors the uniqueness and magnitude of each participant voice.

Each focus group and interview with patients with hip OA and their family caregivers was coded independently by 2 coders (WW, MC, SM, or a research assistant). All of the coders were trained by SM, MW and WM before conducting any coding. Then, the assigned codes were compared by 2 coders and discrepancies were discussed with SM, MW, and WM. The first codebook was developed after the first 3 focus groups and refined after coding the remaining interviews.

The semi-structured interviews with patients with knee OA followed a similar approach as described above. The main coders for this sample were HR and a researcher conducting her master project at the time (PH). SM, WCM, and JMR supervised the coding procedure. Briefly, HR and PH created the initial set of codes after coding the first 4 interviews and then discussed the codes with SM and JMR. Then they created the first draft of the codebook. The codebook was refined as the remaining semi-structured interviews were coded.

### Developing themes

The hybrid approach of using both thematic and content analyses influenced theme development as researchers counted the codes to identify the most commonly mentioned items in development of the codebook, combined with thorough examination of transcripts for unique participant perspectives. To create the final themes, 2 coders (SM and HR) compared the codes from each of the 2 samples and then developed a set of unique themes that encompassed the codes from both samples.

## Results

### Participants

Participants consisted of 46 patients with hip OA (49-85 years old, preoperative n = 20), 16 family caregivers (10 women, 39-82 years old) and 14 patients with knee OA (55-80 years old, preoperative n = 5) who were waiting for TJR surgery or had at least 1 TJR surgery. Most patients were women (n = 40) and native English speakers (n = 64), and the remaining reported Hungarian (n = 4), German (n = 5), Portuguese (n = 1), Persian (n = 1), and French (n = 1) as their first language). The mean age for the study sample was 66.62 years.

### Themes

Analyses revealed 3 main themes: 1. “easier to understand” emphasizes patients’ preferences on delivery formats and features; 2. “what does that mean?” highlights requests for clear and simple information; and 3. “Preparation, right?” shows patients’ perspectives on the best time to have access to the eHealth tool.

#### Theme 1. “Easier to understand.”

This theme emphasizes participants’ preferences on different delivery formats and other features that they think eHealth programs should have. Participants provided opinions about written formats, the use of images and videos, access to a voiceover option, including a quiz in the tool, and additional resources and expressed a need for a combination of formats to make learning the material easier for a variety of learning preferences.

##### Written materials

We first presented the information online in written format during both the interviews and focus groups. There was general agreement among participants regarding the usefulness of written materials. While some people indicated the presentation of information in written format is sufficient, for example, *“I love the reading part,”* others indicated *“Just the text isn’t good”* [woman, 76, family caregiver]. Participants further elaborated that written materials did not clearly present information and they still had uncertainties, for example, *“No, I don’t know what 90 degrees was”* [woman, 59, postoperative, hip OA]. And “*I can only assume, if they don’t speak English, they’re not, can’t read English that well either”* [man, 67, postoperative, knee OA].

##### Images

Most participants in all the groups liked images, for example, *“I’d like, I’d like visuals. I mean that’s, that’s pretty basic information”* [woman, 61, postoperative, hip OA]. In general, our participants believed images are better than written materials and are more clear, for example, “*it just reinforces it, it shows you a picture of what could happen to your knee”* [woman, 78, postoperative, knee OA] or *“The images I think are better because the images would reach a wide variety of people versus the words”* [woman, 55, preoperative, hip OA] or *“Easier to understand, as far as the words”* [woman, 54, postoperative, hip OA]. Participants also mentioned they would like images to be taken in a real environment with an actor compared to video-scribe style animated videos for example, *“The, real life is better. I, I just find the scribbling detracts from the information.”* [woman, age not reported, preoperative, hip OA].

##### Voiceovers

Participants in both hip, knee and family caregiver groups opinions regarding using voiceovers were mixed. The mock-ups included slides with voiceovers which highlighted the key points on the page, and participants were asked to comment on the concept of a voiceover rather than the content of what was said. The voiceovers in the mock-up played automatically when participants went to the next page. Some indicated the voiceovers *“are very good”* and *“reinforces”* what they have learned by texts and images. One participant also mentioned *“I might use the audio. It’s like hearing somebody, it’s, it’s like having this kinda discussion”* [woman, 59, preoperative, hip OA]. Other participants did not prefer the voiceovers and expressed their feelings regarding voiceovers as *“kill the voiceover, kill it”* and *“I don’t do well with people yacking at me from that”* [woman, 76, postoperative, knee OA]. Although, most participants were not against the idea of including the voiceovers in an online tool, they recommended voiceovers should not be played automatically, such as *“I didn’t mind the voice in there, um, I think you had, should be able to have of course the option of having it or not having it”* [woman, 73, postoperative, hip OA].

##### Videos

All participants had positive opinions regarding use of videos in our online modules, for example, “*I think this format for me would work well, I mean when you were doing the video*” [man, 72, postoperative, knee OA] and *“That for me is, that just explains it in a nutshell. And you can, and so visually you hear it, you see what not to do. So that is, like I said before, videos for me work.”* [woman, 49, preoperative, hip OA]. Most participants had positive opinions regarding the videos that were taken in a real environment (ie, non-animated videos) and used an actor for delivering the information, such as “*I think the real person is fantastic”* [man, 72, preoperative, hip OA]. However, participants’ opinions about whiteboard videos were often not positive and considered them as being *“too busy”* and *“too fast.”* Others also indicated “*it’s really irritating”* and *“it tends to be a little bit jerky”* [woman, 77, postoperative, knee OA] and *“I was paying attention [to] the drawing. Cause I really wasn’t paying attention to the message that’s being delivered”* [woman, 59, postoperative hip OA]. Despite the many negative comments, a few participants in each group expressed positive opinions regarding the whiteboard video. For example, 1 participant mentioned, *“The whiteboard one for me, was a kind of um, more fun and more interesting, and if I had shown up and I was sleepy, and my brain fully wasn’t here, that one might be a little more light-hearted, then encourage me to look at it.”* [man, 70, preoperative, hip OA].

##### Quizzes

Participants disclosed negative attitudes toward using quizzes, for example *“Don’t throw quizzes at me, just give me statements”* [man, 83, preoperative hip OA], and “*if your question is about a quiz my answer is that it doesn’t interest me. I have enough anxiety with. . .without having to deal with a quiz and failing it* [woman, 79, postoperative, knee OA]. While fewer in number, there were positive attitudes toward quizzes, indicating that taking quizzes can reinforce learning or test their understanding, suggested by the comment: *“I think giving them an opportunity to give feedback, in terms of how much of the information they took in, I think it is a good idea”* [man, 60, postoperative, knee OA]. Another participant added they *“think the exercise is a good thing, I think it just reinforces like, did I get that point”* [woman, 40, family caregiver]. Specifically, participants with hip OA mentioned that taking quizzes should be optional, suggested in this example *“Well, it could be an option right. You could get to the end and say ‘check yourself, are you going to be safe.’ Something like that”* [man, 73, postoperative, hip OA] and *“quite often there’s this kind of quiz at the end, and I don’t usually go through them because I’ve just assumed that if I’ve read the chapter well, then I don’t need to be tested on it”* [woman, 77, postoperative, knee OA].

Based on participants’ perspectives, yes and no quizzes (or true/false) were the most favorable type of quizzes mainly because they were *“simple”* and *“fast.”* Participants in the hip groups expressed only positive attitudes toward multiple choice quizzes while some participants in the knee group expressed negative opinions as well for example, *“It’s kind of like it’s trying to trick you. That’s my first reaction”* [woman, 64, postoperative, knee OA]. While fewer participants commented on ordering quizzes whereby participants select the correct order of statements, it seems that the opinions regarding this type of quiz were mixed. For instance, some participants found them fun to do, for example, *“Yup it’s playful. Yes, it makes you think”* [woman, 59, postoperative, hip OA], while others found them not interesting, for example, *“I don’t think I would wanna be spending my time shifting these things into the right order”* [woman, 76, postoperative]. Interestingly, drag and drop quizzes raised a large number of negative comments in all the groups such as *“It’s almost insulting our intelligence, it’s almost too simple”* [woman, 40, family caregiver] and *“I don’t see the value of it”* [woman, 79, postoperative, knee OA]. However, there were a few participants who considered this type of quiz as *“fun.”*

##### Additional materials

Participants in all the groups were asked to comment on having a resource page at the end of each module. We received positive comments regarding this idea, for example, *“Most definitely, I actually think it’s better than. . . I’m actually starting to develop of a file folder at home with all the papers. And like it would’ve been—it would be nice if it was all in one place”* [woman, 55, preoperative, hip OA]. Participants mentioned that they would also prefer to have a review page and a checklist page at the end of the online tool.

In summary, this theme reveals that participants were in agreement that although written materials are generally beneficial to include, having images and videos enriches the delivery. However, participants strongly preferred real-life images and videos compared to animated visuals. When considering formats within a prehabilitation module, having optional material such as quizzes and voiceover is preferred. A resource page was determined to add value to the module and participants appreciated the additional information and resources.

#### Theme 2. “What does that mean?”

This category highlights requested characteristics of the eHealth tool for delivering prehabilitation education. Participants spoke about the importance of emphasizing key information, providing clear rationale using simple language, and how the information needs to be relevant to their needs.

##### Emphasizing

One of the most important characteristics of the online module mentioned only by individuals with hip OA was the necessity of emphasizing the vital information regarding hip precautions, such as *“when there’s a huge sentence saying ‘do not do these things or you will be at risk of dislocating your new hip.’ For me, that’s enough.”* [woman, age not reported, preoperative, hip OA]. This point was not mentioned by patients with knee OA.

##### Be clear and simple

Participants in the hip group indicated that the information should be delivered in a *clear and simple* way, as was described by a participant who stated *“and even hip precautions, like somebody who is ESL [English as a second language], like what does that mean? I know hip precautions is the word you use, but to me it might be. . . and I’m just saying this because of the fact that I do have the adult ed [education] background and it’s always simple, simpler is better.”* [woman, 55, preoperative, hip OA]. Similarly, participants in the knee OA group felt clear examples using real people were best, for example, *“real person, talking to you, explaining it. Like I think the messaging is very clear”* [man, 60, postoperative, knee OA].

##### Providing reasons

Both hip and knee participants also mentioned that they would like to hear about the *reasons* for doing or not doing certain activities. They mentioned that knowing the reasons for doing certain types of activities help them to understand and remember those activities better. It also increases the chances of following the recommendations, for example, *“No, why don’t you bend past 90? [another participant: Yea, why?]. Why.”* [man, 57, preoperative, hip OA] and *“see but they don’t tell you why. If you have something that showed you this is, is the correct correspondence, and you know this is the right thing to do and explain why. I think that’s a good way to do it”* [woman, 78, postoperative, knee OA].

##### Be *relevant*, personalized, and customizable

Participants in both hip and knee groups mentioned that they prefer to receive *relevant information* to their health condition. It seems that in many educational sessions, participants received information about several health conditions that were not necessarily related to them (eg, information related to hip and knee replacements were presented together). In addition, participants also mentioned that they would prefer *relevant examples* and they criticized the ones that might be less relevant to their condition. One participant reflected the examples were too specific to some populations and not others, stating *“Like I could see that golf would be difficult for someone who’s in their 80s. You know the golfers pick up, the picture right there”* [woman, 59, postoperative, hip OA]. Another participant emphasized that in terms of finding relevant information “*everybody’s different in that respect. Some people are great at hunting down that kind of information and some people are not”* [man, 67, postoperative, knee OA]. Furthermore, participants also wanted the opportunity to *customize and personalize the content* based on their needs, that is, if they find specific information/sections important or more relevant to them, they should have the option to save that information to be able to review it when needed. This was best described by the following examples: *“It’d be nice, I don’t know. Securing a way that’s something personal that you could cut and paste, and, and customize, and pictures that you need currently”* [woman, 59, postoperative, hip OA] and *“well convenience you can do it at your leisure, when it fits your schedule. And you can just read it and re-read it as many times as you want to”* [woman, 64, postoperative, knee OA].

Theme 2 highlights the importance of the module to provide information in a way that is easy to understand and that clearly highlights the vital information with rationale for the importance. Having material that is customizable/personalized was preferred so that participants were able to save the information most relevant to their needs and review it at a later time.

#### Theme 3. “Preparation, right?”

This theme provides an overview of participants’ perspectives on the best time to have access to the online tool providing the information related to their health condition. Participants identified the importance of having access to prehabilitation education within a time frame that allowed adequate time for review and consolidation of the information.

##### As soon as possible

Most participants mentioned that they would like to receive the education and information related to their health condition as soon as they are informed they need the surgery (in most cases the wait time before the surgery was between 6 to 12 months). Knowing the information early enough could help them to be prepared financially, be able to obtain the necessary equipment, and make the required environmental modifications (eg, in their homes and cars). An illustrative example, 1 participant shared, *“My doctor sent me for an x-ray and she came back and said ‘hey, you need a hip replacement!’ That’s when I want it, right from, even a year before ‘cause it, I need the financials to build up.”* [woman, 59, postoperative, hip OA]. Participants also felt having enough time was important to physically prepare for surgery, for example, 1 participant explained *“yeah I think it’s much better, especially as I said earlier about the kinds of physical activity that’s beneficial before you have the surgery. Preparation, right?”* [woman, 77, postoperative, knee OA].

##### Close to the surgery date

Some participants mentioned that receiving the information several weeks to 1 month before their surgery is enough. The main reason for not wanting to receive the information earlier was the possibility of forgetting the information, for example, *“The further away the surgery, the less information I would think. So, if you didn’t have time to really think and dwell on it, for me, I’m putting myself in their—a month ahead would be plenty just cause you know, it’s a done deal and you need to this and this and this and this and this and this, you just roll with it.”* [woman, 66, postoperative, hip OA].

Theme 3 suggests that patients vary in their preference for when they obtain prehabilitation educational material, as some prefer to review it long in advance of their surgery while others prefer to review it right before surgery. As such, providing access to prehabilitation earlier will allow those who want to review it right away to do so, while those wanting to wait to a time closer to surgery can save it for later and review it when they decide to.

## Discussion

The current qualitative study provides information on patient and caregiver preferences and perspectives regarding delivery formats, features, and characteristics of an eHealth tool aiming to deliver prehabilitation education for patients undergoing THR and TKR surgery. As we anticipated, the views were similar across the groups. The mean age of participants in our study was 66.62 years, which aligns with the age of those receiving TKR or THR, 65.7% of whom are 65 years or older.^2^ The findings showed that participants had positive comments about almost all delivery (eg, text, images, videos) formats. However, most participants preferred a combination of written materials, images and videos presented in the online mock-up. This is consistent with studies that show using images and animations when providing health care education to adult patients is more effective than using written materials, and can enhance information retention and recall, comprehension, and satisfaction with care.^23-26^ All participants had positive attitudes toward videos and most believed voiceovers could be added but should be optional rather than automatically starting. Interestingly, our participants indicated preference for images and videos taken and recorded in real environments. They mentioned these types of images and videos represented their condition and life environment better than the animated ones. Specifically, most participants were opposed to the animated (video scribe) video and found it distracting and too fast. This was in contrast to the findings of other studies that found animated videos more effective and non-threatening than live-action videos.^27,28^ In a study with minority groups, George et al^29^ found a preference toward animated videos over live-action ones. Their participants indicated that they could relate to animated videos more than live-action ones in which they felt that the actors might be “inauthentic.” This difference in preference for live-action and animated videos might be a result of the participants’ age in our study (eg, 49-85 years old), as the mean age in George’s study was 39 years old. Older adults may prefer live-action and classic video styles more than animated ones, perhaps because they are more familiar with them. The diversity of the male and female actors in the pictures and videos should also be considered, as race, culture, race, geographical location, education level, and language likely play a role in how patients will perceive the education.

Similarly, while some participants indicated no issue with taking quizzes, others expressed strong negative views. Specifically, most participants did not prefer game-type quizzes (eg, ordering) as they considered them *“childish.”* However, they were not against the idea of having optional and simple quizzes (eg, true and false). Quizzes and assessments are vital for learning and are recommended to be integrated into eHealth tools.^30,31^ Although they could be included as voluntary tasks as suggested by the participants, patients should be encouraged to test their knowledge by taking the quizzes (eg, by showing a progress bar, giving certificates, or online badges).

Participants mentioned several main characteristics for eHealth tools. They indicated that important information should be emphasized, be simple and clear, provide reasons, and be relevant, personal and customizable. The requested characteristics were in line with guideline recommendations related to increasing health literacy and developing educational materials for older adults.^32,33^ Older adults often experience a decline in cognitive abilities with reductions in information processing speed, working memory, attention span and drawing conclusions.^34,35^ Therefore, educational materials for this patient population should be (1) simple, direct and explicit; (2) have examples and be explanatory; (3) have repetitions and emphases; and (4) focus on what is important and be limited only to the information that patients need.^32^ These recommendations are further addressed in Table 2.

**Table 2.** Recommendations for creation of an eHealth module for delivery of presurgical education.

Formats, features and characteristics	Recommendation
Combining delivery formats	Offering mixed media including written, images and videos for a range of learning preferences of the audience
Audio	The voice over audio recordings should be an optional feature which do not automatically play
Video	Videos including actors/actresses rather than animated or cartoon videos are more appropriate
Quizzes	Optional and simple quizzes at the end of the module for knowledge review and reinforcement
Customizable	Education materials should be relevant to the diagnosis and personalized wherever possible
Rationale	There should be clear explanations and rationale for why the information is important to know, with examples throughout the module
Access timeline	The eHealth module should be offered to patients as soon as possible, and can be reviewed as close to surgery as desired
Repetition and emphasis	Key points and critical reminders should be repeated throughout the module as appropriate (eg, following hip precautions)
Critical page	A page that highlights the critical points for the patient to be aware of (eg, precautions, what to avoid)

Most participants in our study indicated that they would like to have access to the eHealth tool as soon as they find out they are having THR and TKR surgery. Studies show that patients start searching for health information related to their health concern even before their initial visit with their physician.^36-39^ Curiosity, dissatisfaction with the care, having unanswered questions, and health-related anxiety, motivate patients to search for information, especially online.^34^ Participants in our study expressed concerns about the information being of good quality and trustworthy. These findings are supported by other studies regarding eHealth education for chronic conditions, whereby participants reported concerns about accessibility, privacy, and “potentially confusing interfaces.”^35^ Considering that reliability of online information is one of the main challenges that patients face,^40^ it is important to facilitate and provide access to reliable information.

As previously indicated, participants with hip or knee OA, and family caregivers expressed similar preferences regarding formats, features, and characteristics of an eHealth tool. The main observed difference between the hip and knee OA groups was the hip OA group discussed the importance of emphasizing key points regarding the hip precautions. In contrast, the knee OA group did not mention this as a vital factor in online education. This difference can be due to the nature of the precautions, for example, some surgeons advise patients not bend their hip past 90° for as long as 3 months to minimize the risk of dislocation and subsequent need for surgical revision. However, knee precautions tend to focus on twisting and kneeling movements, which are easier for patients to limit in their day-to-day functioning.

The following limitations should be considered when interpreting the findings from this study. First, developing and interpreting the codes cannot be without researchers’ biases. However, we attempted to mitigate this bias by including researchers from different backgrounds (eg, psychology, physiotherapy, occupational therapy). Moreover, participants in this study were from the silent generation (1928-1945) and baby boomer (1946-1964), therefore, it is expected that their preferences are different from other age groups and our findings cannot be used for developing tools for these other groups. In addition, most participants in our study were able to work with computers and were familiar with the concept of eHealth tools, which may not be the case for all older adults. Future studies should consider participant literacy with devices they use to access eHealth, as this is an important variable in the access and uptake of online education.^41^ Finally, only family caregivers of patients with hip OA were recruited for this study, this may restrict the transferability the findings to family caregivers of patients with knee OA.

In the current study there were participants who used third person language to describe some challenges in using eHealth tools. We did not clarify whether these participants encountered these challenges themselves or had heard these from others. These statements that mention others’ challenges should be considered when interpreting and generalizing the findings.

Despite these limitations, this study is unique in its aim to capture both patients’ and caregivers’ perspectives on formats, features, and characteristics of an eHealth tool that provides educational materials for patients undergoing THR and TKR. Despite our study being aimed at informing the development of an eHealth tool, some participants voiced that they would still want to have human interaction. This is a vital point that should be remembered by researchers developing eHealth tools, as well as health care providers and health organizations who are supporting these tools. That is, regardless of their quality, eHealth tools should *not* be a substitute for human interactions.

## Conclusion

The current study aimed to address the gap in literature on the perspectives of both patient and caregivers regarding prehabilitation education using eHealth technologies for those undergoing THR and TKR surgery. Our study involved patients and caregivers by showing a mock-up before developing the eHealth tool, which is the next step of our project. Providing this mock-up made it easier for participants to comment on different formats, features, and characteristics of an eHealth tool. When developing eHealth tools, the present data suggests the importance of providing a combination of written and visual material that is simple, clear and accessible. Our findings indicate that patients should be given access to prehabilitation as early as possible, so that they start learning the education based on their preferred time. In addition, patients should be able to select and customize the information based on their needs prehabilitation. Future work should focus on integrating both patient and caregiver perspectives early in the development of prehabilitation education tools such as eHealth and online methods.
